# Therapeutic choices and disease activity after 2 years of treatment with cladribine: An Italian multicenter study (CladStop)

**DOI:** 10.1111/ene.16250

**Published:** 2024-03-28

**Authors:** Irene Schiavetti, Alessio Signori, Angela Albanese, Jessica Frau, Eleonora Cocco, Lorena Lorefice, Sonia di Lemme, Roberta Fantozzi, Diego Centonze, Doriana Landi, Girolama Marfia, Elisabetta Signoriello, Giacomo Lus, Chiara Zecca, Claudio Gobbi, Rosa Iodice, Leonardo Malimpensa, Cinzia Cordioli, Diana Ferraro, Francesca Ruscica, Livia Pasquali, Anna Repice, Paolo Immovilli, Maria Teresa Ferrò, Simona Bonavita, Massimiliano Di Filippo, Gianmarco Abbadessa, Flora Govone, Maria Pia Sormani, CladStop study group, Gianmarco Abbadessa, Angela Albanese, Simona Bonavita, Martina Cardi, Emanuele Cassano, Diego Centonze, Eleonora Cocco, Antonella Conte, Cinzia Cordioli, Massimiliano Di Filippo, Sonia Di Lemme, Elena Di Sabatino, Roberta Fantozzi, Diana Ferraro, Maria Teresa Ferrò, Jessica Frau, Carolina Gabri Nicoletti, Claudio Gobbi, Flora Govone, Luigi Grimaldi, Paolo Immovilli, Rosa Iodice, Doriana Landi, Luigi Lavorgna, Lorena Lorefice, Giacomo Lus, Leonardo Malimpensa, Girolama Marfia, Giuseppina Miele, Francesca Napoli, Livia Pasquali, Anna Repice, Francesca Ruscica, Irene Schiavetti, Alessio Signori, Elisabetta Signoriello, Carmine Siniscalchi, Maria Pia Sormani, Stefano Tozza, Francesca Vitetta, Chiara Zecca

**Affiliations:** ^1^ Department of Health Sciences University of Genoa Genova Italy; ^2^ Centro Sclerosi Multipla Ospedale Binaghi Cagliari Azienda Sanitaria Locale (ASL) Cagliari Cagliari Italy; ^3^ Dipartimento Scienze Mediche e Sanità Pubblica Università di Cagliari Cagliari Italy; ^4^ Unit of Neurology Istituto di Ricovero e Cura a Carattere Scientifico (IRCCS) Neuromed Pozzilli Italy; ^5^ Department of Systems Medicine Tor Vergata University Rome Italy; ^6^ Multiple Sclerosis Clinical and Research Unit, Department of Systems Medicine Tor Vergata University Rome Italy; ^7^ Centro Sclerosi Multipla, II Clinica Neurologica Università della Campania Luigi Vanvitelli Naples Italy; ^8^ Multiple Sclerosis Center, Neurocenter of Southern Switzerland EOC Lugano Switzerland; ^9^ Faculty of Biomedical Sciences Università della Svizzera Italiana Lugano Switzerland; ^10^ Clinica Neurologica DSNRO Università Federico II di Napoli Napoli Italy; ^11^ Mediterranean Neurological Institute Neuromed Istituto di Ricovero e Cura a Carattere Scientifico (IRCCS) Pozzilli Italy; ^12^ Centro Sclerosi Multipla Azienda Socio Sanitaria Territoriale (ASST) Spedali Civili di Brescia Montichiari Italy; ^13^ Department of Neurosciences, Ospedale Civile di Baggiovara Azienda Ospedaliero‐Universitaria di Modena Modena Italy; ^14^ Unità operativa di Neurologia Fondazione Istituto G.Giglio Palermo Italy; ^15^ Neurology Unit, Department of Clinical and Experimental Medicine University of Pisa Pisa Italy; ^16^ Department of Neurology 2 Careggi University Hospital Florence Italy; ^17^ Neurology Unit, Emergency Department Guglielmo da Saliceto Hospital Piacenza Italy; ^18^ Neurological Unit, Cerebrovascular Department, Neuroimmunology, Center for Multiple Sclerosis ASST Crema Crema Italy; ^19^ Dipartimento di Scienze Mediche e Chirurgiche Avanzate Università della Campania Luigi Vanvitelli Naples Italy; ^20^ Clinica Neurologica, Dipartimento di Medicina e Chirurgia Università di Perugia Perugia Italy; ^21^ I Division of Neurology University of Campania "Luigi Vanvitelli" Naples Italy; ^22^ Centro Sclerosi Multipla–Neurologia di Mondovì Cuneo Italy; ^23^ IRCCS Ospedale Policlinico San Martino Genoa Italy

**Keywords:** cladribine, efficacy, multiple sclerosis, real‐world data, safety, treatment response

## Abstract

**Background and purpose:**

Cladribine tablets, a purine analogue antimetabolite, offer a unique treatment regimen, involving short courses at the start of the first and second year, with no further treatment needed in years 3 and 4. However, comprehensive evidence regarding patient outcomes beyond the initial 24 months of cladribine treatment is limited.

**Methods:**

This retrospective, multicenter study enrolled 204 patients with multiple sclerosis who had completed the 2‐year course of cladribine treatment. The primary outcomes were therapeutic choices and clinical disease activity assessed by annualized relapse rate after the 2‐year treatment course.

**Results:**

A total of 204 patients were enrolled; most patients (75.4%) did not initiate new treatments in the 12 months postcladribine. The study found a significant reduction in annualized relapse rate at the 12‐month follow‐up after cladribine completion compared to the year prior to starting therapy (0.07 ± 0.25 vs. 0.82 ± 0.80, *p* < 0.001). Furthermore, patients with relapses during cladribine treatment were more likely to start new therapies, whereas older patients were less likely. The safety profile of cladribine was favorable, with lymphopenia being the primary registered adverse event.

**Conclusions:**

This study provides insights into therapeutic choices and disease activity following cladribine treatment. It highlights cladribine's effectiveness in reducing relapse rates and disability progression, reaffirming its favorable safety profile. Real‐world data, aligned with previous reports, draw attention to ocrelizumab and natalizumab as common choices after cladribine. However, larger, prospective studies for validation and a more comprehensive understanding of cladribine's long‐term impact are necessary.

## INTRODUCTION

In recent years, there has been a radical change in the therapeutic scenario of multiple sclerosis (MS). The new knowledge of the disease has enabled the development of new disease‐modifying therapies (DMTs), with different mechanisms of action, greater efficacy, and different safety and tolerability conditions. The current generation of DMTs include a wide range of immune modulators, immune depletion, and repopulating agents [1].

Such treatment comprises cladribine tablets, a small purine analogue antimetabolite that mimics adenosine, inhibits the action of the adenosine deaminase enzyme, and causes an interim reduction of lymphocytes with predominance in B cell and T cell counts followed by reconstitution of adaptive functions [2].

In 2017, cladribine was licensed by the European Medicines Agency as the first oral pulsed therapy for the treatment of adult patients with highly active relapsing MS [3].

Each tablet contains 10 mg cladribine, and the recommended total dose is 3.5 mg/kg body weight administered through two short courses at the beginning of the first and second year. Following completion of the two courses, no further cladribine treatment is required in years 3 and 4, but it is not contraindicated (summaries of product characteristics (SmPCs) Mavenclad April 2022). During the clinical trials, repeating the dose routinely beyond year 2 was not associated with significantly improved disease control (SmPCs Mavenclad April 2022) [4].

This treatment‐free window offers different opportunities compared to continuous therapy regimens, such as patients' flexibility in family planning and attenuating or active vaccine administration [5], albeit real‐world data confirm a normal humoral and cell‐mediated immune response to vaccination following cladribine treatment regardless of the time of application and the lymphocyte count [6, 7].

Different phase IV studies are ongoing aimed at collecting new evidence for further characterizing cladribine tablets' benefit/risk ratio.

Several postapproval data are arising from international real‐world cohorts and have well characterized the efficacy and safety of the first 2 years of treatment with cladribine tablets [8, 9, 10].

Furthermore a post hoc analysis of the CLARITY study already supported few years ago the continuation of treatment in year 2 even if a patient reported clinical disease activity in year 1, as most of them experienced benefit from treatment dosing completion [11].

However, the evidence on the follow‐up at 2 years of treatment with cladribine tablets meant as the end of 24 months and beyond is limited.

This analysis aims to investigate the therapeutic choices and clinical disease activity of patients who completed a 2‐year course with cladribine.

## METHODS

### Study design and data collection

This is a multicenter, retrospective observational study that enrolled patients with MS who had completed 2 years of treatment with cladribine, with an observational follow‐up period of at least 6 months. The baseline for this study is defined as the completion of 2 years of treatment with cladribine, which corresponds to 24 months from the therapy start and is referred to as “CladStop” in this study.

Demographic characteristics, information about comorbidities and prior infections, and data related to the patients' MS history were collected. Additionally, clinical, laboratory, and magnetic resonance imaging (MRI) variables (in terms of gadolinium enhancing lesions and new T2 lesions) obtained during the 2 years of treatment with cladribine were retrieved from medical charts. These variables include lymphocyte count, Expanded Disability Status Scale (EDSS) scores, MRI lesion counts, and relapse information. Data on the therapies initiated after the 2‐year course of cladribine treatment were also collected.

MRI data, relapse occurrences, and changes in EDSS scores were collected at 6 and 12 months after CladStop. The evaluation of disability progression was considered in terms of an increase of at least 1 point on the EDSS if the baseline EDSS was equal to or less than 5.5, or 0.5 points if the EDSS was greater than 5.5.

Any relevant adverse events that occurred from the start of treatment with cladribine were documented in the electronic case report form.

Study data were collected and managed using REDCap electronic data capture tools hosted at University of Genoa [12]. REDCap (Research Electronic Data Capture) is a secure, web‐based software platform designed to support data capture for research studies, providing (i) an intuitive interface for validated data capture, (ii) audit trails for tracking data manipulation and export procedures, (iii) automated export procedures for seamless data downloads to common statistical packages, and (iv) procedures for data integration and interoperability with external sources [13].

### Outcomes

The primary outcomes of this study are the therapeutic choices and clinical disease activity assessed by the annualized relapse rate (ARR) following the completion of the 2‐year treatment course with cladribine.

The choice of ARR as the primary outcome is supported by its widespread use as an important measure in clinical trials evaluating the efficacy of treatments for MS [14, 15], such as cladribine. These trials have shown promising results, with a significant decline in relapse rates observed after the initiation of therapy [16, 17].

### Statistical analysis

Continuous variables were described using mean and SD or median and range or interquartile range; categorical variables were reported as counts and percentages.

The time to initiate a new treatment after CladStop was assessed using a Kaplan–Meier curve and analyzed using a multivariable Cox regression model, which was adjusted for baseline characteristics.

Differences in ARR over time were determined with Friedman test and a post hoc pairwise comparison using the Bonferroni correction.

Significance level was set at 5% for all analyses, and all tests were two‐tailed.

All analyses were performed with SPSS (IBM SPSS Statistics, version 24.0; IBM, Armonk, NY, USA).

### Ethics statement

The study was conducted in compliance with the principles of the Declaration of Helsinki. The protocol was approved by the regional ethics committee (CER Liguria: 273/2022—DB id 12,395, 20 June 2022). Written informed consent was obtained from all participants before starting any study procedures.

## RESULTS

A total of 204 people with MS (pwMS) from 17 Italian and one Swiss center were enrolled between June 2022 and May 2023. Demographic and clinical characteristics are shown in Table 1.

**TABLE 1:** Demographic and clinical characteristics (*N* = 204).

Sex, females, *n* (%)	144 (70.6%)
Age, years, mean (SD)	36.6 (10.45)
BMI, kg/m^2^, mean (SD)	25.1 (4.81)
Ethnicity, Caucasian, *n* (%)	204 (100.0%)
MS duration, years, mean (SD)	8.2 (7.45)
Last EDSS assessed prior to initiation of therapy, median [interquartile range]	2.0 [1.0–3.0]
Total number of previous DMTs, median [range]	1.0 [1.0–6.0]
Last previous DMT, *n* (%)	
Dimethyl fumarate	54 (29.2%)
No previous DMT	28 (15.1%)
Interferon	26 (14.1%)
Glatiramer acetate	24 (13.0%)
Fingolimod	22 (11.9%)
Natalizumab	10 (5.4%)
Teriflunomide	10 (5.4%)
Rituximab	6 (3.2%)
Ocrelizumab	3 (1.6%)
Alemtuzumab	1 (0.5%)
Daclizumab	1 (0.5%)
At least one relapse in past 12 months, *n* (%)	128 (62.7%)
Number of relapses in past 12 months, median [range]	1.0 [0.0–4.0]
Presence of MRI activity within the past 12 months, *n* (%)	136/164 (82.9%)
Presence of at least one GEL within the past 12 months, *n* (%)	97/162 (59.9%)
Number of GELs within the past 12 months, median [range]	1.0 [0.0–2.0]

Abbreviations: BMI, body mass index; DMT, disease‐modifying therapy; EDSS, Expanded Disability Status Scale; GEL, gadolinium enhancing lesion; MRI, magnetic resonance imaging; MS, multiple sclerosis.

The mean age at CladStop was 36.6 (SD = 10.45) years with a range of 18 to 68 years and a predominance of female patients (70.6%). Approximately three quarters of the patients (76.0%) had no comorbidities (Table 2).

**TABLE 2:** Comorbidities and screening for latent infections (*N* = 204).

Presence of at least one comorbidity	49 (24.0%)
Major depressive disorder	10 (4.9%)
Hypertension	3 (1.5%)
Coronary heart disease	3 (1.5%)
Malignant tumor	3 (1.5%)
Cerebrovascular disease	2 (1.0%)
Chronic liver disease	2 (1.0%)
Diabetes	2 (1.0%)
Chronic kidney disease	0 (0.0%)
HIV	0 (0.0%)
HBV	0 (0.0%)
Hematological disease	0 (0.0%)
Other	32 (15.7%)
Presence of tuberculosis assessed prior to initiation of therapy	2 (1.0%)
Presence of hepatitis C assessed prior to initiation of therapy	1 (0.5%)
Presence of hepatitis B assessed prior to initiation of therapy	0 (0.0%)

*Note*: Data are presented as *n* (%).

Abbreviations: HBV, hepatitis B virus; HIV, human immunodeficiency virus.

Before starting cladribine treatment, patients had a median EDSS score of 2.0 (with an interquartile range of 1.0–3.0), and on average, they had undergone one (range = 0–6) previous DMT (the most common was dimethyl fumarate [DMF], 29.2%). Most patients (*n* = 128, 62.7%) experienced at least one relapse in the 12 months before starting cladribine, and 136 individuals presented MRI activity (data available on 164 pwMS).

The median observation period after CladStop was 17.5 months, ranging from 6 (minimum allowed) to 36 months. At 12 months, the probability of starting a new treatment was 12.1%, which increased to 24.6% at 24 months.

**FIGURE 1:**
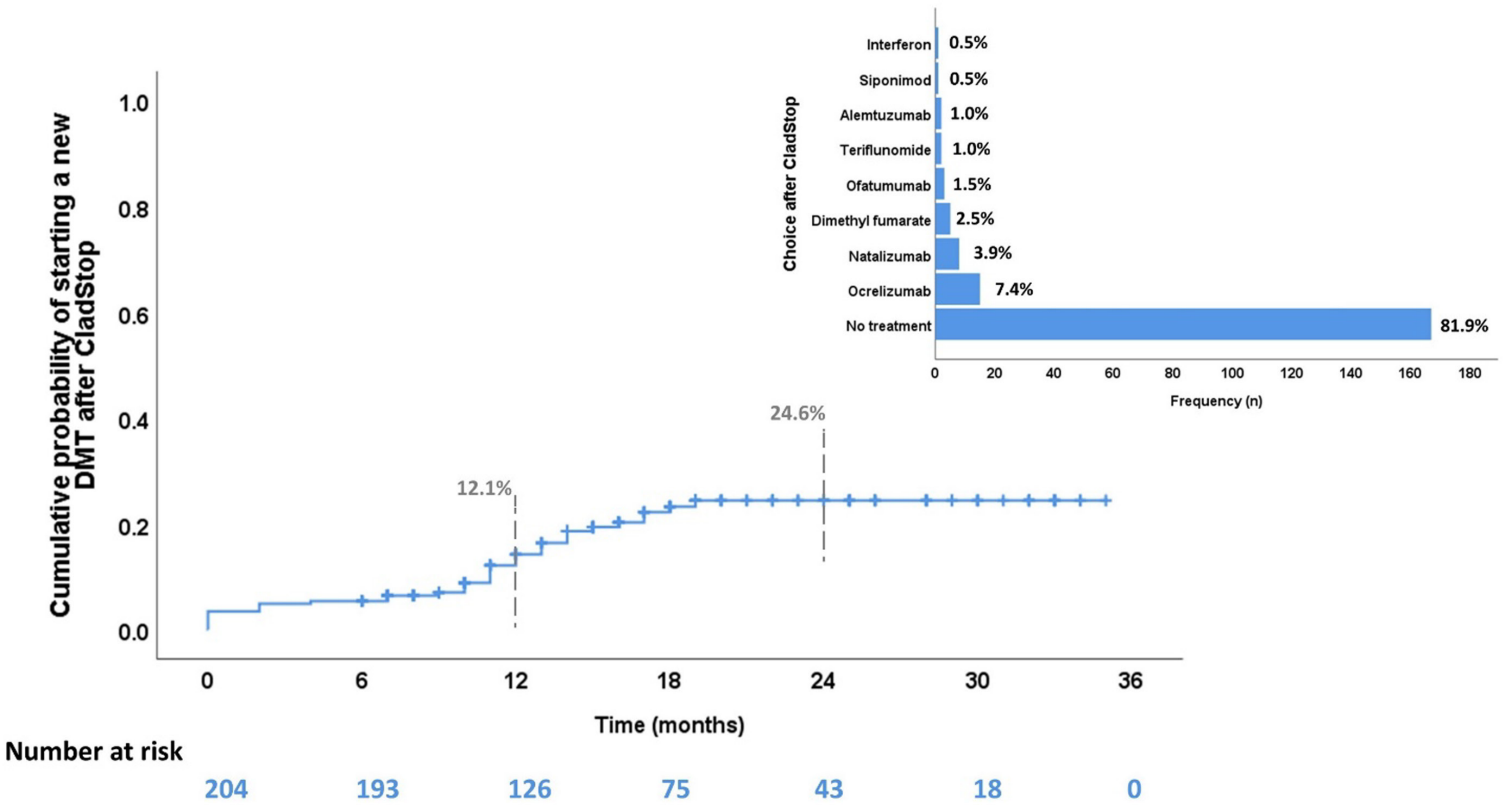
Therapeutic choice after CladStop. DMT, disease‐modifying therapy.

Among all participants, 37 individuals (18.1%) started a new treatment following cladribine therapy. Table 3 presents the characteristics of these patients. The most common treatments were ocrelizumab (*n* = 15, 7.4%) and natalizumab (*n* = 8, 3.9%). No patients received an additional cycle of cladribine during the entire follow‐up as per Italian rules (Figure 1).

**TABLE 3:** Characteristics of patients who initiated a new treatment after 2 years of treatment with cladribine.

Sex	Time to start, months	Age, years	Comorbidities	Previous DMT	Disease duration, yrs	EDSS prior to cladribine	EDSS during first year of treatment	EDSS during second year of treatment	Relapses in past 12 months	Relapses during cladribine treatment	Previous MRI activity	MRI activity during treatment
*Ocrelizumab*												
Female	12	27	Yes	No previous DMT	2	2.0	2.0	2.0	Yes		Yes	
Female	10	43		Fingolimod	17	1.0	2.0	2.0			Yes	
Female	11	24	Yes	Glatiramer acetate	4	1.0	1.0	2.0	Yes		Yes	Yes
Female	11	46		Dimethyl fumarate	9	5.0	3.5	6.0	Yes	Yes	Yes	Yes
Male	14	35		Natalizumab	9	2.5	2.5	2.5				Yes
Male	2	31		Dimethyl fumarate	3	2.0	2.0	1.5	Yes		Yes	Yes
Female	11	31	Yes	Dimethyl fumarate	13	2.0	1.5	1.5	Yes	Yes	Yes	Yes
Female	14	28	Yes	No previous DMT	2	1.0	1.0	1.0			Yes	Yes
Female	10	23		Dimethyl fumarate	5	1.0	1.0	1.0	Yes	Yes	Yes	Yes
Female	11	22		Fingolimod	7	1.0	1.0	1.0		Yes	Yes	Yes
Female	18	25		Dimethyl fumarate	6	3.0	2.0	2.0	Yes			Yes
Female	0	30		No previous DMT	2	1.5	1.5	0.0	Yes	Yes		Yes
Female	0	62		Interferon	7	3.0	3.0	2.5	Yes	Yes	Yes	Yes
Male	0	28		Dimethyl fumarate	4	1.0	1.0	1.0	Yes		Yes	Yes
Female	0	27		Fingolimod	5	0.0	0.0	1.0		Yes	Yes	Yes
*Natalizumab*												
Female	2	24		Interferon	5	0.0	0.0	0.0				
Female	14	33		Interferon	2	1.0	1.0	1.0	Yes		Yes	
Female	19	17		No previous DMT	2	0.0	0.0	2.0	Yes		Yes	Yes
Female	4	36		Dimethyl fumarate	10	1.0	1.0	1.0	Yes	Yes	Yes	Yes
Female	0	30		Fingolimod	8	1.0	0.0	0.0	Yes		Yes	Yes
Female	12	26	Yes	Fingolimod	5	2.0	2.0	2.0			Yes	Yes
Female	0	33		Fingolimod	18	1.0	1.0	1.0		Yes	Yes	Yes
Female	0	28	Yes	Glatiramer acetate	6	1.0	2.0	2.0	Yes		Yes	Yes
*Dimethyl fumarate*												
Male	7	47		No previous DMT	2	2.0	1.0	2.0	Yes		Yes	Yes
Female	10	29		No previous DMT	2	1.0	1.0	1.0	Yes		Yes	
Female	12	41		Teriflunomide	11	2.5	1.5	1.0	Yes	Yes	Yes	Yes
Male	13	36		Dimethyl fumarate	10	2.0	1.0	1.0	Yes		Yes	
Female	17	35		No previous DMT	2	2.0	1.0	1.0	Yes		Yes	
*Ofatumumab*												
Female	13	24		Dimethyl fumarate	3	1.0	1.5	2.0	Yes	Yes	Yes	Yes
Female	17	48		Dimethyl fumarate	8	5.5	5.5	6.0	Yes		Yes	
Female	2	52	Yes	Glatiramer acetate	21	3.0	4.0	4.0				
*Teriflunomide*												
Female	15	45	Yes	Interferon	4	2.0	2.0	3.0	Yes		Yes	Yes
Female	9	24		Interferon	12	1.0	0.0	0.0	Yes		Yes	
*Alemtuzumab*												
Female	13	23		Teriflunomide	6	1.0	1.0	1.0				
Female	16	24	Yes	Natalizumab	6	1.0	1.0	1.0		Yes		
*Siponimod*												
Female	11	27		Glatiramer acetate	6	6.0	6.0	6.0		Yes		
*Interferon*												
Female	7	20		Natalizumab	2	1.5	2.0	1.5		Yes	Yes	Yes
Overall	9.1 ± 6.04	32.0 ± 9.97	9/37 (24.3%)		6.6 ± 4.78	1.0 [1.0–2.0]	1. 0 [1.0–2.0]	1.5 [1.0–2.0]	24/37 (64.9%)	14/37 (37.8%)	33/37 (89.2%)	24/37 (64.9%)

Abbreviations: DMT, disease‐modifying therapy; EDSS, Expanded Disability Status Scale; MRI, magnetic resonance imaging.

Table 4 shows the analysis of time to starting a new treatment after CladStop. Patients who experienced relapses during the 2 years of cladribine treatment had a higher probability of starting a new therapy after CladStop (hazard ratio [HR] = 3.78, 95% confidence interval [CI] = 1.82–7.85, *p* < 0.001) compared to patients without relapses. Furthermore, the probability of starting a new therapy decreased with the age of the patients (HR = 0.68, 95% CI = 0.47–0.98, *p* = 0.039). Patients who had higher disease activity before cladribine, as indicated by MRI activity or previous relapses, did not exhibit a significantly higher probability of initiating new therapy compared to other patients.

**TABLE 4:** Factors associated with starting a new therapy after CladStop.

Parameter	Univariate HR (95% CI); *p*	Multivariate HR (95% CI); *p*
Sex, males vs. females	0.32 (0.13–0.83); 0.019	0.44 (0.17–1.16); 0.10
Age, 10 years	0.60 (0.42–0.88); 0.008	0.68 (0.47–0.98); 0.039*
MRI activity in the previous 12 months	1.65 (0.58–4.70); 0.35	–
Presence of relapses in the previous 12 months	1.12 (0.57–2.21); 0.74	–
MS duration, years	0.97 (0.92–1.02); 0.23	–
Presence of relapses during cladribine treatment	5.23 (2.68–10.22); <0.001	3.78 (1.82–7.85); <0.001*
Last EDSS during cladribine treatment	0.89 (0.73–1.10); 0.28	–
Presence of MRI activity during cladribine treatment	1.99 (1.01–3.91); 0.046	1.29 (0.63–2.67); 0.49

*Note*: Cox regression model. * indicates statistically significant.

Abbreviations: CI, confidence interval; EDSS, Expanded Disability Status Scale; HR, hazard ratio; MRI, magnetic resonance imaging; MS, multiple sclerosis.

At 12 months after CladStop, 40 patients (28.0% among those with this minimum follow‐up) showed an improvement of at least 0.5 points on the EDSS, whereas 18.8% reported a disability progression. Of these, 70.4%, still at 12 months, had neither MRI activity nor relapses.

Nineteen patients showed MRI activity (data not shown).

**FIGURE 2:**
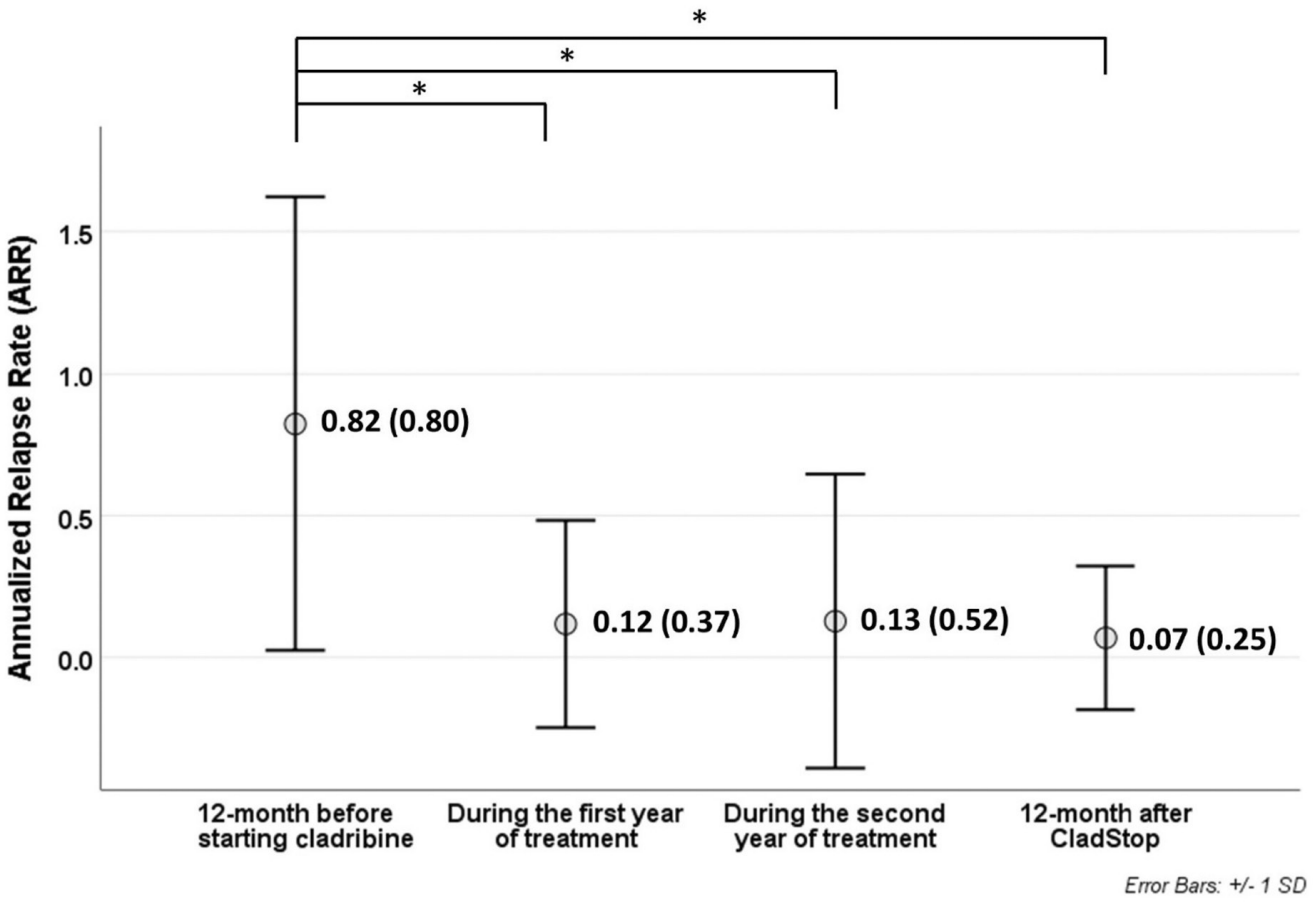
Annualized relapse rate before, during, and after cladribine. * indicates statistically significant.

The ARR improved significantly as early as the first year of treatment (0.12 ± 0.37, *p* < 0.001), confirmed after the second year (0.13 ± 0.52, *p* < 0.001); the ARR further decreased after 12 months of follow‐up after CladStop (0.07 ± 0.25, *p* < 0.001), again compared with the ARR in the year prior to cladribine start (0.82 ± 0.80; Figure 2).

**FIGURE 3:**
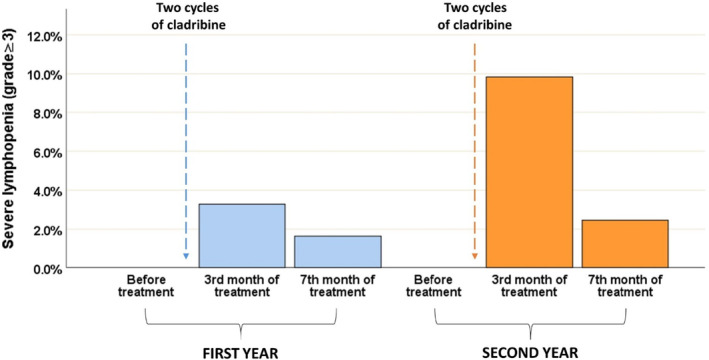
Lymphocyte count during 2 years of treatment.

In terms of lymphocyte counts, the trends over the 2 years mirror each other, with a marked increase in lymphopenia (grade III or higher) immediately after the treatment cycle (3.6% in the first year and 8.8% in the second year), followed by a recovery at month 7 (1.8% in the first year and 2.6% in the second year). This trend is particularly noticeable in the second year (Figure 3). The presence of high grade of lymphopenia was consistent among all patients throughout the entire study period, regardless of their age, except during the third month of treatment in the second cycle, when a higher frequency of events was observed in the elderly group (>50 vs. ≤50 years old: 26.3% vs. 6.2%, *p* = 0.004).

Eighteen (8.8%) patients presented at least one adverse event (AE) for a total of 23 cases. Twenty AEs occurred in 15 patients during the 2 years of treatment and three in three patients in the 12 months following CladStop. One serious AE, prostate cancer, occurred in one pwMS during the 12‐month following CladStop; this AE was considered not related to the treatment by the treating neurologist (Tables 5 and 6). Three cases of oral herpes, indicated by physician as probably or certainly related to cladribine use, were reported during 2 years of treatment. They were mild and resolved within a few days; these patients did not have lymphopenia. However, it is worth mentioning that prophylaxis for herpes zoster is recommended for patients with grade 4, for grade 3 lymphopenia, or for immunocompromised individuals [18].

**TABLE 5:** Safety data.

		Total	During treatment with cladribine	During 12‐month following CladStop
Patients with at least one AE		18	15	3
Total number of AEs		23	20	3
Severity	Mild	11 (47.8%)	10 (50.0%)	1 (33.3%)
	Moderate	11 (47.8%)	9 (45.0%)	2 (66.7%)
	Severe	1 (4.3%)	1 (5.0%)	0 (0.0%)
Relationship with cladribine	Certainly related	4 (17.4%)	4 (20.0%)	0 (0.0%)
	Definitely related	1 (4.3%)	1 (5.0%)	0 (0.0%)
	Probably related	6 (26.1%)	6 (30.0%)	0 (0.0%)
	Possibly related	6 (26.1%)	5 (25.0%)	1 (33.3%)
	Likely related	0 (0.0%)	0 (0.0%)	0 (0.0%)
	Not related	6 (26.1%)	4 (20.0%)	2 (66.7%)
SAEs		1 (4.3%)	0 (0.0%)	1 (33.3%)

*Note*: Data are presented as *n* (%).

Abbreviations: AE, adverse event; SAE, serious AE.

**TABLE 6:** Listing of adverse events.

System organ class (SOC)	Preferred terms (PT)	Severity	Relationship with cladribine
*During treatment with cladribine*			
General disorders and administration site co	Pyrexia	Mild	Probably related
Infections and infestations	Influenza	Mild	Not related
Infections and infestations	Nasopharyngitis	Mild	Probably related
Infections and infestations	Fungal infection	Mild	Probably related
Infections and infestations	Oral herpes	Mild	Probably related
Infections and infestations	Oral herpes	Mild	Certainly related
Infections and infestations	Oral herpes	Mild	Certainly related
Skin and subcutaneous tissue disorders	Neurodermatitis	Mild	Not related
Skin and subcutaneous tissue disorders	Eczema herpeticum	Mild	Possibly related
Skin and subcutaneous tissue disorders	Pityriasis	Mild	Possibly related
Skin and subcutaneous tissue disorders	Psoriasis	Moderate	Probably related
Investigations	Platelet count decreased	Moderate	Not related
Gastrointestinal disorders	Abdominal pain upper	Moderate	Not related
Gastrointestinal disorders	Pharyngitis	Moderate	Possibly related
Nervous system disorders	Writer's cramp	Moderate	Possibly related
Nervous system disorders	Coordination abnormal	Moderate	Possibly related
Immune system disorders	Urticaria	Moderate	Probably related
Vascular disorders	Hypotension	Moderate	Certainly related
Blood and lymphatic system disorders	Lymphopenia	Severe	Certainly related
Infections and infestations	Vulvovaginal candidiasis	Moderate	Definitely related
During 12 months following CladStop			
Pregnancy, puerperium, and perinatal conditions	Pregnancy	Mild	Not related
Infections and infestations	Gingivitis	Moderate	Possibly related
Neoplasms benign, malignant, and unspecified	Prostate cancer	Moderate	Not related

## DISCUSSION

This study reports on the therapeutic choice at the end of 2 years of treatment, the evidence of cladribine's effectiveness in reducing relapse rates and disability progression, and the drug's safety profile.

In general, the results from this study overall demonstrate an overall significant improvement in the ARR at the 12‐month follow‐up after CladStop compared to the pretreatment period and the first 2 years of treatment. This observation suggests a concrete reduction in the occurrence of relapses in pwMS irrespective of their treatment choice after the completion of the second cycle of cladribine.

The analysis of time to starting a new treatment after CladStop reveals two significant associations (both *p* < 0.001) and provide insights into the factors influencing treatment decision following 2 years of treatment with cladribine. First, patients who experienced relapses during the 2 years of cladribine treatment demonstrated a threefold higher probability of initiating a new therapy after CladStop. Second, there is an inverse proportional relationship between patients' age and the likelihood of starting a new therapy after CladStop. However, most of the patients did not initiate new treatments during the 12 months following the completion of the treatment with cladribine, suggesting a stable clinical status that did not require subsequent additional therapies. Only a small proportion of patients (9%) showed MRI activity and/or worsening on the EDSS (18.8% in 143 patients with evaluation at 12‐month follow‐up).

Overall, cladribine showed a favorable safety profile consistent with its mechanism of action [19]; lymphopenia (grade ≥ 3) was reported in a subgroup of patients immediately after each cycle of cladribine, both during the first year (about 4%) and during the second year (about 9%). Lymphocyte counts returned to a normal range as we moved away from the last dose (at the seventh month of treatment), consistent with data from the literature [2].

Noticeably, two of three cases of oral herpes occurred in nonelderly adult patients coming from a previous DMF treatment, whereas one was not previously treated. This aligns with recent literature [20], which reported that herpes infections predominantly occurred in patients who had received DMF as prior therapy.

After CladStop, only three AEs were recorded in three patients. Among these, only one case of moderate severity gingivitis was reported as possibly related to cladribine and occurred 7 months after 2 years of treatment.

These results are consistent with other reports based on real‐life data, confirming that ocrelizumab and natalizumab are the primary choices for switching after 2 years of treatment with cladribine [21]. Additionally, they indicate a reduction in ARR during and after treatment compared to the pretreatment period, as well as a predominance of mainly mild to moderate adverse events [16]. Finally, they demonstrate a similar rate of improvement in EDSS after the end of 2 years of treatment [22].

This study has limitations. To begin with, it is important to note that the study is observational and retrospective. There is a possibility that physicians might not have accurately reported specific details, and the lack of clinical evaluations at certain time points could affect the overall reliability of the findings.

Additionally, the chosen follow‐up period after the completion of 2 years of cladribine treatment is relatively short (a minimum of 6 months and a maximum of 36 months). A longer term follow‐up would provide a more comprehensive understanding of the sustained effectiveness and safety profile of cladribine beyond the immediate posttreatment period.

Finally, the study primarily focuses on clinical outcomes, such as relapse rates and disability progression, but does not extensively explore or collect information about patient preferences or factors influencing their subsequent therapeutic choices. A more comprehensive understanding of patient perspectives, for example, through patient‐reported outcomes, could enhance the interpretation of treatment decisions.

## CONCLUSIONS

Evidence from these real‐world data suggests predictors for starting a new treatment after 2 years of cladribine treatment and confirms the efficacy of cladribine on disease activity and on disability worsening (with a consistent reduction on ARR and an improvement on the EDSS).

The incidence of AEs was relatively low, with no treatment‐related serious AEs reported. However, the retrospective design of this study together with the selection of a limited Italian–Swiss cohort of patients could bias these results, and further studies with larger sample sizes and longer follow‐up would be useful to validate these findings.

## AUTHOR CONTRIBUTIONS

**Irene Schiavetti:** Conceptualization; writing – original draft; writing – review and editing; methodology; formal analysis; project administration; supervision. **Alessio Signori:** Methodology; writing – review and editing; supervision; conceptualization; project administration. **Angela Albanese:** Writing – review and editing; writing – original draft. **Jessica Frau:** Writing – review and editing; investigation; data curation. **Eleonora Cocco:** Data curation; investigation; writing – review and editing. **Lorena Lorefice:** Data curation; investigation; writing – review and editing. **Sonia di Lemme:** Data curation; investigation; writing – review and editing. **Roberta Fantozzi:** Data curation; investigation; writing – review and editing. **Diego Centonze:** Writing – review and editing; investigation; data curation. **Doriana Landi:** Data curation; investigation; writing – review and editing. **Girolama Marfia:** Data curation; investigation; writing – review and editing. **Elisabetta Signoriello:** Data curation; investigation; writing – review and editing. **Giacomo Lus:** Data curation; investigation; writing – review and editing. **Chiara Zecca:** Data curation; investigation; writing – review and editing. **Claudio Gobbi:** Data curation; investigation; writing – review and editing. **Rosa Iodice:** Data curation; investigation; writing – review and editing. **Leonardo Malimpensa:** Data curation; investigation; writing – review and editing. **Cinzia Cordioli:** Data curation; investigation; writing – review and editing. **Diana Ferraro:** Data curation; investigation; writing – review and editing. **Francesca Ruscica:** Data curation; investigation; writing – review and editing. **Livia Pasquali:** Data curation; investigation; writing – review and editing. **Anna Repice:** Data curation; investigation; writing – review and editing. **Paolo Immovilli:** Data curation; investigation; writing – review and editing. **Maria Teresa Ferrò:** Data curation; investigation; writing – review and editing. **Simona Bonavita:** Data curation; investigation; writing – review and editing. **Massimiliano Di Filippo:** Data curation; investigation; writing – review and editing. **Gianmarco Abbadessa:** Data curation; investigation; writing – review and editing. **Flora Govone:** Data curation; investigation; writing – review and editing. **Maria Pia Sormani:** Validation; supervision; writing – review and editing; methodology; conceptualization; project administration.

## CONFLICT OF INTEREST STATEMENT

Abbadessa Gianmarco personal compensation from Janssen and Merck for traveling and/or advisory boards. Albanese Angela is an employee of Merck Serono S.p.A., Rome, Italy, an affiliate of Merck KGaA, Darmstadt, Germany. Bonavita Simona was Speaker and/or AB honoraria from Novartis, BMS, Merck Serono, Biogen, Janssen Cilag, Alezio, Horizon, Roche. Centonze Diego is an advisory board member of Almirall, Bayer Schering, Biogen, GW Pharmaceuticals, Merck Serono, Novartis, Roche, Sanofi‐Genzyme and Teva and has received honoraria for speaking or consultation fees from Almirall, Bayer Schering, Biogen, GW Pharmaceuticals, Merck Serono, Novartis, Roche, Sanofi‐Genzyme and Teva. He also is the principal investigator in clinical trials for Bayer Schering, Biogen, Merck KGaA (Darmstadt, Germany), Mitsubishi, Novartis, Roche, Sanofi‐Genzyme and Teva. His preclinical and clinical research was supported by grants from Bayer Schering, Biogen Idec, Celgene, Merck Serono, Novartis, Roche, Sanofi‐Genzyme and Teva. Cocco Eleonora serves on scientific advisory boards and received honoraria for speaking from Alexion, Biogen, BMS, Janssen, Merck, Novartis, Roche, and Sanofi Genzyme. Cordioli Cinzia received Honoraria for travelling or speaking in advisory Board from Biogen, Novartis, Merck Serono, Roche, BMS. Di Filippo Massimiliano participated on advisory boards and steering committees for and received speaker or writing honoraria, research support and funding fortravelling from Alexion, BMS, Bayer, Biogen Idec, Genzyme, Horizon, Merck, Mylan, Novartis, Roche, Siemens Healthineers, Teva and Viatris. Fantozzi Roberta received honoraria or consultation fees from Roche, Novartis, Merck Serono, BMS. Ferraro Diana has received travel grants and/or fees for speaking and/or advisory boards from Alexion, Biogen, Bristol‐Myers Squibb, Celgene, Merck, Novartis, Roche, Sanofi. Frau Jessica serves on scientific advisory boards for Biogen, Merck, Genzyme, Novartis, and has received honoraria as a speaker from Merck, Biogen, Novartis, Genzyme, TEVA, Alexion. Gobbi Claudio received consulting fees, or research grants from Almirall, Biogen Idec, Bristol Meyer Squibb, Lundbeck, Merck, Novartis, Sanofi, Teva Pharma, Roche. (Ente Ospedaliero Cantonale (employer) received compensation for Gobbi Claudio's speaking activities). Govone Flora received grants from Roche. Immovilli Paolo received fees for speaking or advising from Roche, Biogen, Sanofi, Bristol Squibb Meyers, Novartis and Merck. Iodice Rosa reports personal fees from Merck, Biogen, Teva, Sanofi Genzyme, Roche, Almirall, Viatris. Landi Doriana received travel funding from Biogen, Merck‐Serono, Sanofi‐Genzyme, Teva, speaking or consultations fees from Sanofi‐Genzyme, Merck‐Serono, Teva, Biogen, Roche; Research sponsorship from Roche. Lorefice Lorena received honoraria for consultancy or speaking from Biogen, Novartis, Sanofi, Genzyme, Serono and Teva and Almirall. Lus Giacomo received personal compensation for activities with Biogen Idec, Merck Serono, Novartis, Sanofi‐Aventis Pharmaceuticals, Teva neuroscience as a consultant and speaker and received research support from Biogen Idec, Merck Serono, and Novartis. Marfia Girolama Alessandra received speaking or consultation fees from Almirall, Bayer‐Schering, Biogen, Genzyme, Merck‐Serono, Novartis, Teva, Sanofi‐Genzyme. Pasquali Livia received personal compensations for speaking or consultancy from Sanofi, Novartis, Merck, Alexion, Biogen; and supporting for attending meetings from Sanofi, Merck. Repice Anna Maria has received honoraria for speaking and for participating to advisory board from Merck, Biogen‐Idec, Sanofi‐Genzyme, Novartis, Roche, Bristol Mayer. Schiavetti Irene received consulting fees from Horizon, Hippocrates Research, Eyepharma, Hoya Holding N.V., DMG Italia and DreamsLab. Signori Alessio received consulting fees from Horizon, Chiesi and Sanofi‐Genzyme outside of this work. Signoriello Elisabetta received personal compensation from Almirall, Biogen, Genzyme, Novartis, and Teva fortraveling and advisory boards. Sormani MP received consulting fees from Roche, Biogen, Merck, Novartis, Sanofi, Celgene, Immunic, Geneuro, GSK, Medday; received payment or honoraria for lectures, presentations, speakers' bureaus, manuscript writing or educational events from Roche, Biogen Merck, Novartis, Sanofi, Celgene; participated on a Data Safety Monitoring Board or Advisory Board for Roche, Sanofi, Novartis, Merck. Zecca Chiara received consulting fees, or research grants from Almirall, Biogen Idec, Bristol Meyer Squibb, Lundbeck, Merck, Novartis, Sanofi, Teva Pharma, Roche [Ente Ospedaliero Cantonale (employer) received compensation for Zecca Chiara's speaking activities]. Other authors have nothing to disclose.

## Data Availability

The data that support the findings of this study are available from the corresponding author upon reasonable request.

## APPENDIX A CladStop Study Group

**Table:**

Abbadessa, Gianmarco	I Division of Neurology–University of Campania “Luigi Vanvitelli,” Naples, Italy
Albanese, Angela	Department of Health Sciences, University of Genoa, Genoa, Italy
Bonavita, Simona	Dipartimento di Scienze Mediche e Chirurgiche Avanzate, Università della Campania Luigi Vanvitelli, Naples, Italy
Cardi, Martina	Department of Biomedical Metabolic and Neurosciences, University of Modena and Reggio Emilia, Modena, Italy
Cassano, Emanuele	Clinica Neurologica, DSNRO Università Federico II di Napoli, Naples, Italy
Centonze, Diego	Unit of Neurology, Istituto di Ricovero e Cura a Carattere Scientifico (IRCCS) Neuromed, Pozzilli, Italy. Tor Vergata University, Department of Systems Medicine, Rome, Italy
Cocco, Eleonora	Centro Sclerosi Multipla Ospedale Binaghi Cagliari, Azienda Sanitaria Locale (ASL) Cagliari, Italy. Dipartimento Scienze Mediche e Sanità Pubblica, Università di Cagliari, Cagliari, Italy
Conte, Antonella	Centro Sclerosi Multipla Policlinico Umberto I, Rome, Italy
Cordioli, Cinzia	Centro Sclerosi Multipla, Azienda Socio Sanitaria Territoriale (ASST) Spedali Civili di Brescia, Montichiari, Italy
Di Filippo, Massimiliano	Clinica Neurologica, Dipartimento di Medicina e Chirurgia Università di Perugia, Perugia, Italy
Di Lemme, Sonia	Unit of Neurology, IRCCS Neuromed, Pozzilli, Italy
Di Sabatino, Elena	Clinica Neurologica, Dipartimento di Medicina e Chirurgia Università di Perugia, Perugia, Italy
Fantozzi, Roberta	Unit of Neurology, IRCCS Neuromed, Pozzilli, Italy
Ferraro, Diana	Department of Neurosciences, Ospedale Civile di Baggiovara, Azienda Ospedaliero–Universitaria di Modena, Modena, Italy
Ferrò, Maria Teresa	Neuroimmunology, Center for Multiple Sclerosis, Cerebrovascular Department, Neurological Unit, Azienda Socio Sanitaria Territoriale (ASST), Crema, Italy
Frau, Jessica	Centro Sclerosi Multipla Ospedale Binaghi Cagliari, Azienda Sanitaria Locale (–ASL) Cagliari, Italy.
Gabri, Nicoletti Carolina	Multiple Sclerosis Clinical and Research Unit, Department of Systems Medicine, Tor Vergata University, Rome, Italy
Gobbi, Claudio	Multiple Sclerosis Center, Neurocenter of Southern Switzerland, EOC, Lugano, Switzerland. Faculty of Biomedical Sciences, Università della Svizzera Italiana, Lugano, Switzerland
Govone, Flora	Centro SM–Neurologia di Mondovì, Mondovì (Cuneo), Italy
Grimaldi, Luigi	Fondazione Giglio di Cefalù, Cefalù (Palermo), Italy
Immovilli, Paolo	Neurology Unit, Emergency Department, Guglielmo da Saliceto Hospital, Piacenza, Italy
Iodice, Rosa	Clinica Neurologica, DSNRO Università Federico II di Napoli, Naples, Italy
Landi, Doriana	Multiple Sclerosis Clinical and Research Unit, Department of Systems Medicine, Tor Vergata University, Rome, Italy
Lavorgna, Luigi	I Division of Neurology–University of Campania “Luigi Vanvitelli,” Naples, Italy
Lorefice, Lorena	Centro Sclerosi Multipla Ospedale Binaghi Cagliari–ASL Cagliari, Cagliari, Italy
Lus, Giacomo	Centro Sclerosi Multipla, II Clinica Neurologica, Università della Campania Luigi Vanvitelli, Naples, Italy
Malimpensa, Leonardo	Mediterranean Neurological Institute Neuromed (IRCCS), Pozzilli, Italy
Marfia, Girolama	Multiple Sclerosis Clinical and Research Unit, Department of Systems Medicine, Tor Vergata University, Rome, Italy
Miele, Giuseppina	Dipartimento di Scienze Mediche e Chirurgiche Avanzate, Università della Campania Luigi Vanvitelli, Naples, Italy
Napoli, Francesca	Multiple Sclerosis Clinical and Research Unit, Department of Systems Medicine, Tor Vergata University, Rome, Italy
Pasquali, Livia	Department of Clinical and Experimental Medicine, Neurology Unit, University of Pisa, Pisa, Italy
Repice, Anna	Department of Neurology 2, Careggi University Hospital, Florence, Italy
Ruscica, Francesca	Fondazione Giglio di Cefalù, Cefalù (Palermo), Italy
Schiavetti, Irene	Department of Health Sciences, University of Genoa, Genoa, Italy
Signori, Alessio	Department of Health Sciences, University of Genoa, Genoa, Italy
Signoriello, Elisabetta	Centro Sclerosi Multipla, II Clinica Neurologica, Università della Campania Luigi Vanvitelli, Naples, Italy
Siniscalchi, Carmine	Clinica Neurologica, DSNRO Università Federico II di Napoli, Naples, Italy
Sormani, Maria Pia	Department of Health Sciences, University of Genoa, Genoa, Italy. IRCCS Ospedale Policlinico San Martino, Genoa, Italy
Tozza, Stefano	Clinica Neurologica, DSNRO Università Federico II di Napoli, Naples, Italy
Vitetta, Francesca	Department of Neurosciences, Ospedale Civile di Baggiovara, Azienda Ospedaliero–Universitaria di Modena, Modena, Italy
Zecca, Chiara	Multiple Sclerosis Center, Neurocenter of Southern Switzerland, EOC, Lugano, Switzerland
